# Dental‐Legal Partnerships: Extending the Medical‐Legal Partnership Model to Dentistry

**DOI:** 10.1111/jphd.70070

**Published:** 2026-08-09

**Authors:** Alexander Testa, Rahma Mungia, Jack Tsai

**Affiliations:** ^1^ School of Public Health University of Texas Health Science Center at Houston Houston Texas USA; ^2^ School of Dentistry University of Texas Health Science Center at San Antonio San Antonio Texas USA; ^3^ National Center on Homelessness Among Veterans US. Department of Veterans Affairs Washington DC USA

**Keywords:** access to dental care, dental‐legal partnership, health‐harming legal needs, medical‐legal partnership, social determinants of health

## 1 Introduction

Oral health is a fundamental component of overall health [1, 2, 3] and is shaped not only by clinical care but also by social determinants of health (i.e., the conditions in which individuals live, work, and play) [4, 5, 6]. A growing body of research indicates that hardships such as food insecurity [6, 7, 8], housing instability [6, 9], lack of insurance coverage [10], and employment instability [11, 12] influence oral health and access to dental care. Although many of these issues are economic in nature, they are often intertwined with underlying civil legal issues, such as unsafe housing conditions, wrongful denial of public benefits, and employment disputes [13]. Civil legal issues that negatively affect domains of health—including oral health—are referred to as health‐related legal needs [14, 15].

To address health‐related legal needs, medical‐legal partnerships (MLPs) have emerged as a model that integrates legal services into health care settings by connecting patients with pro bono legal aid to resolve civil legal issues that undermine health and well‐being [13, 16]. By the end of 2025, nearly 500 MLP programs nationwide were operating in hospitals, community health centers, and clinics [15, 17]. MLPs have also been established to reach various populations, such as veterans [18], individuals experiencing homelessness [19], and victims of violence [20]. Dental settings (i.e., dental programs operated within federally qualified health centers [FQHCs], hospitals, dental schools, community clinics, and private dental practices), however, remain largely absent from the MLP model. Indeed, despite the growth of MLPs in numerous medical settings [15] and a brief reference to their potential relevance for dentistry in the National Institute of Dental and Craniofacial Research's *Oral Health in America* report [3], the authors' search of the literature identified no published descriptions of MLPs in dental settings and only a single description of a pilot program in rural Minnesota [21]. The premise of this commentary is to inform the dental community about the potential value of extending the MLP model to dental care, including integrating dental programs into existing MLPs (e.g., within FQHCs) or by forming new partnerships between dental organizations and civil legal aid providers. We also highlight the need for formal research to address questions about the feasibility and effectiveness of incorporating dental care into MLP models.

## 2 Medical‐Legal Partnerships and Health‐Related Legal Needs

The MLP model began in the late 1990s and has since expanded nationally, with a presence in nearly every state supported by organizations such as the National Center for Medical‐Legal Partnership [15, 17]. The premise of the MLP model is that civil legal issues, such as denial of public benefits or living in unsafe housing conditions, carry downstream consequences for one's health [13, 15, 22]. Many health care providers, including dental care providers, commonly encounter individuals affected by health‐related legal needs, whether or not they are aware of the legal issues their patients face. However, providers often lack the time, expertise, training, and resources to identify and resolve these issues.

Table 1 provides examples of health‐related legal needs across the social determinants of health domains and illustrates how addressing them can influence oral health and access to dental care. For example, a patient may experience denial or termination of public benefits such as Medicaid, the Supplemental Nutrition Assistance Program, or disability benefits. The loss of these benefits can cause an economic shock, generate stress, and require substantial time and effort to navigate complex administrative processes to restore them. Together, these challenges can negatively affect oral health by limiting access to dental care and making it more difficult to maintain recommended oral hygiene practices and adhere to dental treatment plans. Legal aid services can help overcome these challenges by working with patients to appeal benefit denials or enroll in eligible programs.

**TABLE 1:** Application of civil legal services to help address social needs and improve oral health among dental patients.

Social determinant of health (I‐HELP domains)	Examples of health‐related legal needs	How legal services can help	Impact on oral health and dental care
Income	Denial or termination of Medicaid/CHIP or disability benefits Incorrect denial of SNAP/WIC benefits Denial of SSDI or SSI Unlawful denial of unemployment benefits	Appeal denial or termination of public benefits Assist with enrollment in Medicaid, SSI/SSDI, or SNAP/WIC Challenge improper health insurance claim denials Secure disability determinations that provide income support	Increased financial resources improve the ability to afford dental visits, medications, and preventive care. Access to nutrition programs supports healthy diets that reduce the risk of dental caries and other oral diseases. Insurance coverage (e.g., Medicaid) increases access to preventive and restorative dental care.
Housing and utilities	Unsafe housing conditions (mold, pests, lead, poor ventilation) Illegal eviction or housing instability Utility shutoffs affecting water or electricity Landlord's refusal to make health‐related repairs	Enforce housing codes and require landlords to remediate hazardous conditions Provide legal representation in eviction proceedings Secure housing subsidies or vouchers Prevent utility shutoffs or negotiate repayment plans	Stable housing improves the ability to attend dental appointments and complete multi‐visit treatments. Reliable access to utilities supports daily oral hygiene (e.g., brushing, flossing). Reduced environmental hazards lower risks of oral infections and systemic health conditions linked to poor oral health.
Education and employment	Workplace discrimination or wrongful termination Wage theft or unpaid overtime Denial of workplace accommodations for health conditions School discipline or barriers to educational access	Enforce worker protection laws and recover unpaid wages Address employment discrimination claims Secure workplace accommodations for health needs Resolve education access issues and protect student rights	Stable employment increases income to afford dental care and oral hygiene supplies. Employer‐provided health and dental insurance improves access to preventive and restorative services. Higher educational attainment is associated with improved oral health knowledge and dental care utilization.
Legal status	Immigration status barriers affecting eligibility for services Incorrect veteran discharge status limiting benefits Criminal record barriers to employment and housing Errors in credit reports affecting housing eligibility	Assist with immigration applications, asylum claims, or legal status adjustments Upgrade veteran discharge status to restore eligibility for VA benefits Expunge or seal eligible criminal records Resolve credit reporting errors	Legal status improvements can increase access to employment, housing, and public benefits that support overall health. Veterans with corrected discharge status may gain eligibility for VA dental services. Greater economic and social stability improves the ability to obtain regular dental care.
Personal and family stability	Domestic or intimate partner violence Child custody disputes or guardianship issues Lack of child support payments Elder abuse or financial exploitation	Obtain protective orders in cases of domestic violence Secure guardianship or custody arrangements Enforce child support obligations Provide legal protection for vulnerable family members	Reducing exposure to violence can prevent facial trauma and dental injuries. Increased family stability reduces stress and improves adherence to oral hygiene and treatment plans. Child support and family stability improve resources for children's preventive dental care.

*Note:* Table is adapted from the National Center for Medical Legal Partnership I‐HELP Framework: https://medical‐legalpartnership.org/response/i‐help/.

Abbreviations: CHIP = Children's Health Insurance Program; SNAP = Supplemental Nutrition Assistance Program; SSDI = Social Security Disability Insurance; SSI = Supplemental Security Income; VA = Department of Veterans Affairs; WIC = Special Supplemental Nutrition Program for Women, Infants, and Children.

While experimental evidence remains limited, prior studies suggest MLPs provide benefits for patients, including improved health care experiences and utilization [23, 24], physical health [23, 24, 25], mental health [23, 24, 26], reduced stress [24], and less emergency department utilization [24, 26, 27]. MLPs are also efficacious in reducing health‐related legal needs [14]. Patients also benefit from improved knowledge of their legal rights and how to navigate the legal system, and this knowledge can be shared with family members and friends, suggesting that MLPs can offer downstream benefits to the broader community [28, 29]. However, one randomized trial found higher anxiety scores and hospital visits associated with MLP referral [26]. Health systems involved in MLPs also report returns on investment through cost savings and increased reimbursements [25, 28].

For dentistry specifically, MLPs could support dental care providers by addressing nonmedical barriers that undermine treatment plans. Dental providers may also gain a deeper understanding of how social conditions and legal issues affect oral health and become more comfortable discussing unmet social needs when there is a clear place to refer patients for support [30, 31]. Dental professionals experience high levels of stress and burnout [32, 33, 34], driven partly by patients' inability to afford treatment [34]. MLPs may help ease this burden, as one study of 532 medical providers found that MLPs reduced clinician stress and burnout by helping providers address patients' social determinants of health [35].

## 3 Extending the Medical‐Legal Partnership Model to Dental Care

The dental‐legal partnership extends the principles of MLPs to dental settings. We define “dental settings” broadly to include any clinic that offers dental services, such as dental programs within FQHCs and hospitals, dental schools, community clinics, and private dental practices. Thus, a dental‐legal partnership can take multiple paths, including (1) a free‐standing alternative to the MLP that operates within dental clinics, or (2) partnerships with dental clinics to integrate oral health services into existing MLPs. For example, FQHCs account for hundreds of MLPs nationwide [17], and 82% of FQHCs operated dental programs in 2023, serving approximately 6.3 million people [36]. Thus, many organizations already house both an MLP and a dental program within the same organization—although understanding how dentistry is integrated into MLPs operating within FQHCs is not well documented in published research to date. In many cases, we believe dentistry programs are not incorporated into existing MLPs, which have mostly focused on primary care, mental health, and pediatric service lines [14, 23, 28]. Indeed, to the author's knowledge, there are no published reports describing whether these dental programs screen or refer patients through their organization's MLP, whether dental patients have benefited indirectly from co‐located legal services, or what barriers have prevented integration. Documenting and building on these existing arrangements, for example, by adding oral health considerations to MLP screening and by training dental teams to refer patients through established MLP channels, may extend legal services to dental patients while requiring minimal additional legal aid capacity.

For dental organizations without access to an existing MLP, including private and community dental practices and dental school clinics, a new dental‐legal partnership can be formed following the eight steps outlined in prior research [13, 37]. First, the partnership begins with a formal agreement between a dental care organization and a civil legal services provider (i.e., a nonprofit legal aid organization) that specifies the responsibilities of both parties, along with the information‐sharing procedures and patient authorizations necessary to comply with the Health Insurance Portability and Accountability Act (HIPAA) and with client‐lawyer confidentiality and privilege. Second, the partnership identifies the patient population and the civil legal needs to be served. This often includes low‐income patients, who experience a disproportionate burden of health‐related legal needs. Many legal aid organizations also restrict services to clients with incomes below a certain threshold, and some partnerships may specialize in specific civil legal needs (e.g., housing or public benefits) to be most effective and sustainable. Third, the dental practice implements screening procedures to identify patients' health‐related legal needs. Screening typically focuses on the I‐HELP domains (Income, Housing, Employment, Legal status, Personal stability) [38] and can be embedded in existing intake paperwork or electronic records and administered during check‐in or clinical visits; an example screening tool and best‐practice guidance are available from the National Center for Medical‐Legal Partnership: https://medical‐legalpartnership.org/screening‐tool [38, 39]. Fourth, a staffing and referral structure is defined; for example, designated dental clinic staff (such as a front‐office coordinator or community health worker) refer qualifying patients, and the legal aid organization identifies one or more attorneys to work with the clinic. Fifth, civil legal aid attorneys may provide services virtually or on‐site periodically to facilitate coordination. Because the essential elements of MLP fidelity have not yet been rigorously established, different implementation arrangements may be explored. Sixth, training is provided to dental providers and staff to help them recognize civil legal issues and determine when referrals are appropriate [40]. Seventh, the partnership develops secure systems for information sharing, including referral protocols and mechanisms for coordinating care and tracking case outcomes. Eighth, sustaining a dental‐legal partnership requires dedicated funding and infrastructure (i.e., contributions from the dental care organization, legal partners, external grants, or philanthropic sources).

## 4 Potential Challenges

Despite the potential benefits, implementing dental‐legal partnerships poses challenges. A major constraint is that demand for legal aid already far exceeds supply [41]. Thus, adding dental referrals without corresponding legal capacity risks lengthening queues rather than resolving needs. In addition, given the limited time and administrative capacity available to many dental clinics, providers may be reluctant to implement screening and referral processes. Still, integrating brief screening questions into existing intake forms can help minimize workflow disruptions, and connections to legal aid services may ultimately offload work from dental providers and staff. Dental‐legal partnerships located solely within dental practices may also miss the many individuals who do not use dental care. According to the American Dental Association's Health Policy Institute, only 52% of children aged 0–18, 40% of adults aged 19–64, and 51% of adults aged 65 and older had a dental visit in 2022. Across income levels, 57% of individuals with household incomes at or above 400% of the federal poverty level had a visit in 2022, compared with 28.3% of those below 100% [42]. Because health‐related legal needs concentrate among low‐income populations [43], those who stand to benefit most may be least likely to be seen in a dental clinic. Thus, these figures reinforce the potential value of anchoring dental‐legal integration within FQHCs and other safety‐net settings that serve low‐income patients. Finally, individual dental practices may lack the connections or resources to develop and sustain these collaborations. Practice‐based research networks that create communities of dental professionals and researchers can serve as an infrastructure to help identify opportunities, facilitate partnerships, create referral pathways, and coordinate legal services [44].

## 5 Areas for Future Research

To understand the feasibility and potential efficacy of such programs, foundational research is needed, including documenting and examining the extent to which FQHC dental programs have been integrated into their organizations' MLPs, as well as what barriers and facilitators exist in attempts to achieve this integration. In addition, pilot programs within dental settings could provide valuable insights into implementation strategies, patient outcomes, and cost implications. Still, reciprocal interest from the legal services community may vary greatly by region, client population, and existing resource needs. Moving forward, studies, including randomized controlled trials, can investigate whether these partnerships improve access to dental care and oral health, enhance treatment adherence, benefit patients' unmet social and legal needs, and potentially improve other health markers beyond oral health. Given the inherently interdisciplinary nature of these questions, research teams spanning dentistry, medicine, law, social work, and public health, and supported by infrastructure such as dental practice‐based research networks, are well‐positioned to advance this agenda [44].

## Funding

The authors have nothing to report.

## Disclosure

During the preparation of this manuscript, Claude (Anthropic) was used edit and refine the text. The authors reviewed and revised all output and take full responsibility for the content of the publication. The views presented are of the authors, alone, and do not represent the views of the US Department of Veterans Affairs, or any other federal agency.

## Conflicts of Interest

The authors declare no conflicts of interest.

## Data Availability

Data sharing is not applicable to this article as no datasets were generated or analysed during the current study.
